# The Hospital World

**Published:** 1910-12-31

**Authors:** 


### THE HOSPITAL WORLD.

Wm. Armitage, Esq., J.P., has been elected chairman of the Ancoats Hospital, Manchester, in the room of the late S. L. Helm, Esq., and Mr. A. E. Gaddum has been appointed honorary secretary.

Her Majesty the Queen has become patron of the Royal London Ophthalmic Hospital.

The Duchess of Albany has consented to become Lady President of the Kingston-Surbiton, Putney, and Wandsworth district of the League of Mercy, in place of Princess Alexander of Teck.

Most people will remember Mr. John Martineau as Charles Kingsley's pupil, a prominent member, in short, of that group of enthusiasts which in the fifties believed philanthropy could achieve the betterment of England. Latham, the author of *Pastor Pastorum*, was another. Martineau's death, only a few days ago, will stir up a host of memories in the seniors of the last generation. His work for that branch of philanthropy which he held hospitals to be is summed up by a correspondent in the *Times* as follows: "At Ipswich, at Chalfont, and at Eversley he set up homes for training nurses, for teaching the feeble-minded, and for the convalescent treatment of invalid children. Martineau's interests reached beyond home. There is now rising on a hill-slope near Hebron, overlooking the traditional oak-tree of Abraham, a hospital which he presented to the United Free Church of Scotland as a centre for the energies of a medical missionary living in that city and long working at a disadvantage from being unable to receive in-patients." This proves the wide scope over which he and the men of similar inspiration spent their energies and money. They—and John Martineau is a good example—do the pioneer work of social reconstruction, and their memories, like Sir Edwin Chadwick's, form signposts for the historian who sees them as the harbingers of Royal Commissions and Government Boards, on which ultimately is forced the work which the philanthropists were the first to see the need for. The modern hospital owes its development to their spirit, and will not let go their memory even when their work has evolved into a more complex and more centralised phase.

The death is announced of Captain Alfred Hutton, late of the King's Dragoon Guards. Captain Hutton was a famous swordsman and a high authority upon everything which concerned fencing, and he possessed a most unique collection of armour from all periods. Captain Hutton was the last of the five founders of the Central London Throat and Ear Hospital. He was appointed the Chairman of the Committee of that institution at one of the earliest meetings in 1874, and retained that position for over twenty-five years, when he was succeeded by another founder, Mr. George Wallis, whose death we have also had to chronicle this year. By the wish of H.R.H. the Duke of Connaught, patron of the Hospital, Captain Hutton was appointed vice-patron upon his resigning the chairmanship. Captain Hutton took a keen interest in the work of the Hospital with which he was connected for so many years, and his death will come as a personal loss to his colleagues on the Board.

At a special Court of Governors of the Royal Hospitals of Bridewell and Bethlem, Alderman Sir George Faudel-Phillips presiding, Dr. William H. B. Stoddart was appointed resident physician and medical superintendent of Bethlem Hospital in the room of Dr. T. B. Hyslop, who has retired. Dr. Stoddart has been assistant physician at Bethlem since 1898, and for the last three years has lived there as deputy superintendent. Dr. J. G. Porter Phillips succeeds Dr. Stoddart as second medical officer.

The annual dinner and whist drive of the Manchester and District Association of Hospital Secretaries was held at the Albion Hotel, Manchester, on Saturday, December 10, when a large company of guests dined under the presidency of Mr. Walter G. Carnt, F.C.I.S., Manchester Royal Infirmary, president of the Association, who was accompanied by Mrs. Carnt. After dinner an enjoyable whist drive took place, and the guests dispersed at 11 p.m. This Association now numbers among its members secretaries of the principal voluntary hospitals in Lancashire, Derbyshire, and Cheshire. Applications for membership should be made to Mr. Hy. J. Eason, F.C.I.S., hon. secretary and treasurer, Manchester Children's Hospital, Pendlebury, near Manchester.

We very much regret (remarks the *Journal* of the Hospital Saturday Fund) to have occasion to appeal on behalf of an old and esteemed friend of the Council of the Fund, Mr. J. W. Nimmo. He was one of the most regular and zealous members of the Surgical Appliance Committee. As an ordinary member, Honorary Secretary, and Chairman, he retained his interest in the work of this Committee for over twenty years. Recently, owing to ill-health, he was obliged to retire. He was also a member of the Executive Committee for many years. In view of his prolonged services, and his infirmity, some of his old friends have subscribed to the Printers' Pension Fund, and formed a sub-committee for the purpose of securing his election for a Printers' pension. He is being recommended by Mr. G. A. Lewcock, who is so well known at Messrs. Spottiswoode and Co.'s establishment and on the Council of this Fund, and supported by Messrs. Bradbury, Agnew and Co. and their employés, and many other interested friends. The following particulars are culled from the election card: John Watkins Nimmo, aged fifty-seven years, suffering from paraplegia, served his apprenticeship with and worked for Messrs. Bradbury, Agnew and Co., of Bouverie Street, E.C., for thirty-one years. From 1898 to 1910 he conducted a small business on his own account in the Holloway Road, N., which failing health compelled him to give up. The Composing Room Chapel of Messrs. Bradbury, Agnew and Co. resolved: "That this Chapel earnestly recommends the candidature of Mr. Nimmo to the consideration of subscribers, believing his case to be one that calls for every sympathy." His candidature is strongly supported by the Executive and Surgical Appliance Committees of the Sunday Fund. Proxies will be thankfully received by any of his supporters, including Mr. A. W. Davis, 54 Gray's Inn Road, W.C. We feel sure Mr. Nimmo's claim needs only to be made known in the proper quarters for his election to be assured.

Mr. R. J. Gilbert, who recently retired from the post of secretary-superintendent of the West London Hospital, which he held for thirty years, was last week presented with an illuminated address and a cheque by the Duke of Abercorn (Chairman of the board of management) on behalf of the board of management and the medical staff.

The December monthly meeting of the board of management of the Herefordshire General Hospital was busy with more than one important matter, but its first act was to record the death of the institution's old friend Mr. J. U. Caldicott. It was in more than one capacity that Mr. Caldicott's regard for the hospital was shown. Routine work never frightened him, and he had been for many years a most familiar figure at the weekly committee meetings of the board of management. The work of the house committee was shared also by him, and he was also one of the honorary auditors. There must have been a good stream of energy behind such a record of work as this, and many a man who does the regular, unexciting work of the weekly committee meetings of a hospital ward is a more genuine supporter of our hospitals than the large subscriber. Personal service is a very stiff test of a man's enthusiasm, and the Herefordshire General Hospital had in Mr. Caldicott a man who passed it well for many years.

